# Bullous Sweet syndrome with a unique photodistributed pattern^☆^

**DOI:** 10.1016/j.abd.2020.10.018

**Published:** 2022-02-08

**Authors:** Mai Endo, Miyuki Yamamoto, Mikio Ohtsuka, Toshiyuki Yamamoto

**Affiliations:** Department of Dermatology, Fukushima Medical University, Fukushima, Japan

Dear Editor,

A 54-year-old female was admitted to our department, complaining of a 4-day history of up to 40.3 °C fever and cutaneous eruptions on the neck. The patient had been suffering from myelodysplastic syndrome (MDS), for which no drugs, including granulocyte-colony stimulating factor (G-CSF), were administered. Physical examination showed numerous concrescences of blisters clearly localized in the V-neck area (Fig. 1a, b). Painful erythematous nodules were scattered on the cheeks, but the tip of the nose was spared. Furthermore, there was one blister on the right forearm as well as painful erythematous nodules on the upper and lower extremities. She did not have any mucosal lesions or a prior history of recurrent oral aphthae. Laboratory examination showed a white blood cell count of 8,100/μL with 3% Band, 21% Seg, 9% Lym, 7% Mono, and 57% Blast, decreased levels of hemoglobin (8.6 g/dL) and platelets (108,000/μL), and increased levels of C-reactive protein (26.02 mg/dL). Blood culture was sterile. A biopsy specimen from the peripheral edge of the bulla on the neck showed a subepidermal bulla and dense neutrophilic infiltration in the upper to mid-dermis (Fig. 2a, b). Immature myeloid cell infiltration was not detected. Infiltration of histiocytes/histiocytoid cells was not observed, and immunohistological examination showed scattered CD68- and MPO-positive cells which were not prominent. Additionally, another biopsy taken from the lower leg showed infiltration of inflammatory cells, mainly lymphocytes, around the vessels in the septa of the subcutaneous fat tissue (Fig. 2c). Hematological examination and bone marrow biopsy revealed transformation from MDS into acute myelocytic leukemia, and chemotherapy including prednisolone was immediately started in the hematology department. After about one week, all of the bullous lesions were re-epithelialized.

**Figure 1:**
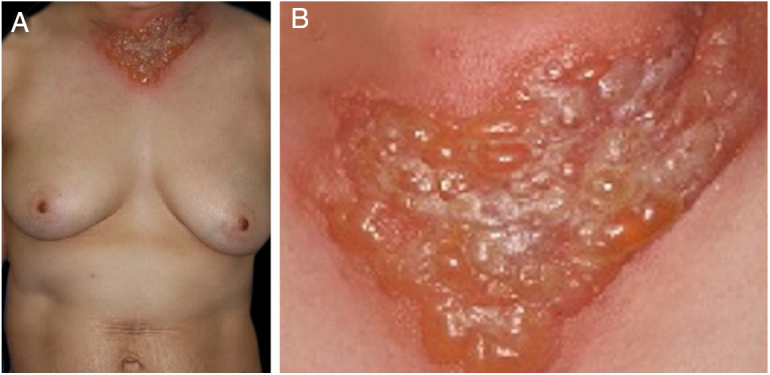
(A), Well-circumscribed, painful, infiltrative erythema with tense blisters in the V-neck area. (B), Close-up view.

**Figure 2:**
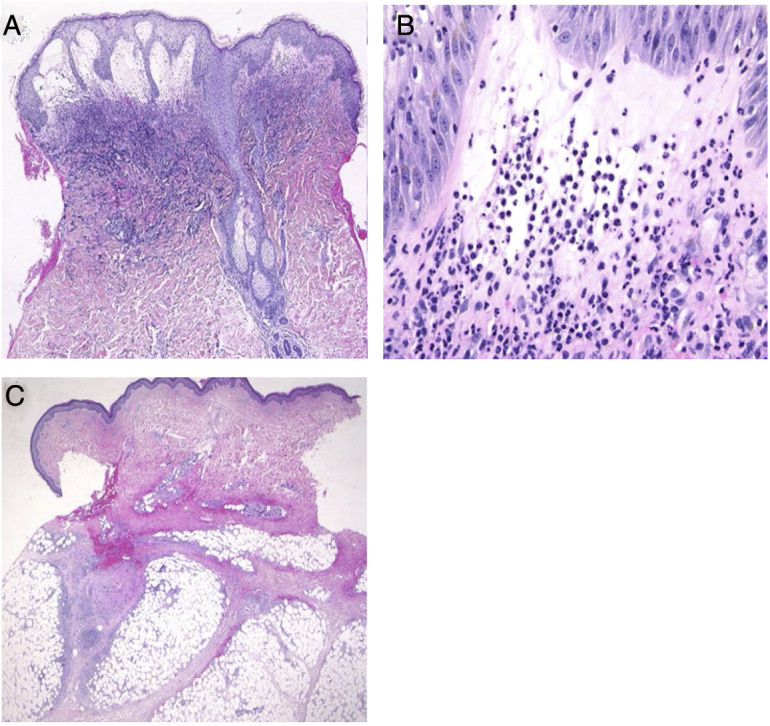
(A), Histological features showing dense neutrophilic infiltration throughout the edematous dermis (Hematoxylin & eosin, original magnification ×40). (B), Higher magnification showing the large number of infiltrating neutrophils (Hematoxylin & eosin, ×400). (C), Histological examination showing a septal panniculitis in the subcutaneous nodule in the lower extremity (Hematoxylin & eosin, ×20).

The present case developed a sudden onset of skin lesions, which histopathologically showed a predominant neutrophilic infiltration without leukocytoclastic vasculitis. In addition, fever, elevated C-reactive protein, and rapid response to systemic steroids were observed. White blood cell count was within the upper limit of the normal range but two times higher than the usual value. Thus, the patient fulfilled the criteria for Sweet syndrome (SS). Cutaneous lesions of SS associated with myelodysplasia tend to be vesicular, bullous, or erosive. The present case developed bullous SS, simultaneously with the blastic crisis of MDS. Of interest, the lesions showed a peculiar distribution of well-circumscribed erythema in the V-neck area not protected by clothes during the season when the dose of ultraviolet radiation is increased in Japan, which was considered to be a photo-Koebner phenomenon. Only several cases of photo-induced or photo-distributed SS have been reported, in which both ultraviolet A and ultraviolet B were candidates for induction of skin lesions.^1^, ^2^, ^3^ Although phototesting was not performed in the present case, the patient had no previous history of photosensitive dermatitis or of taking drugs that can induce photosensitive eruptions. To our knowledge, the current report is the first case of photolocalized bullous SS. The direct action of ultraviolet is speculated to activate and recruit neutrophils via interleukin-8, tumor necrosis factor-α, E-selectin, interleukin-1α, and G-CSF^4^, ^5^ as well as ultraviolet-induced local immunosuppression. Finally, the patient concurrently developed infiltrative erythematous nodules on the face and extremities. Biopsy showed septal panniculitis with lymphocytic infiltration without neutrophil infiltration in either dermis or subcutis; however, all of the concurrent lesions were considered to be part of the same spectrum.

## Financial support

None declared.

## Authors' contributions

Mai Endo: Designed the study; performed the research and contributed to analysis and interpretation of data; wrote the initial draft of the manuscript; read and approved the final version of the manuscript.

Miyuki Yamamoto: Performed the research and contributed to analysis and interpretation of data; read and approved the final version of the manuscript.

Mikio Ohtsuka: Performed the research and contributed to analysis and interpretation of data; read and approved the final version of the manuscript.

Toshiyuki Yamamoto: Designed the study; assisted in the preparation of the manuscript; read and approved the final version of the manuscript.

## Conflicts of interest

None declared.
